# *De novo* Assembly and Analysis of Tissue-Specific Transcriptomes of the Edible Red Sea Urchin *Loxechinus albus* Using RNA-Seq

**DOI:** 10.3390/biology10100995

**Published:** 2021-10-02

**Authors:** Paulette Antiqueo, Rodrigo Zuloaga, Macarena Bastias-Molina, Claudio Meneses, Juan Manuel Estrada, Alfredo Molina, Juan Antonio Valdés

**Affiliations:** 1 Departamento Ciencias Biológicas, Facultad de Ciencias de la Vida, Universidad Andres Bello, Santiago 8370186, Chile; p.antiqueo.a@gmail.com (P.A.); r.zuloaga@uandresbello.edu (R.Z.); macarena.bastias@unab.cl (M.B.-M.); claudio.meneses@unab.cl (C.M.); amolina@unab.cl (A.M.); 2 Interdisciplinary Center for Aquaculture Research (INCAR), Concepción 4030000, Chile; 3 Centro de Biotecnología Vegetal, FONDAP Center for Genome Regulation, Facultad de Ciencias de la Vida, Universidad Andres Bello, Santiago 8370146, Chile; 4 Centro de Investigación Marina Quintay (CIMARQ), Facultad de Ciencias de la Vida, Universidad Andres Bello, Valparaíso 2340000, Chile; mestrada@unab.cl

**Keywords:** edible red sea urchin, *Loxechinus albus*, RNA-seq, reference transcriptome

## Abstract

**Simple Summary:**

Edible red sea urchin (*Loxechinus albus*) is an endemic species of echinoderm distributed along the Chilean coasts. This resource has been overexploited in recent years, depleting their natural populations. At present, there are few reported gene sequences available in public databases, restricting the molecular studies associated with aquaculture for this species. The aim of this study was to present the first annotated reference transcriptome of *L. albus* using NGS technologies and the differential expression transcripts analysis of the evaluated tissues. The transcriptome data obtained in this study will serve as a reference for future molecular research in the edible red sea urchin and other sea urchin species.

**Abstract:**

Edible red sea urchin (*Loxechinus albus*) is an endemic echinoderm species of the Chilean coasts. The worldwide demand for high-quality gonads of this species has addressed the depletion of its natural populations. Studies on this sea urchin are limited, and genomic information is almost nonexistent. Hence, generate a transcriptome is crucial information that will considerably enrich molecular data and promote future findings for the *L. albus* aquaculture. Here, we obtained transcriptomic data of the edible red sea urchin by Illumina platform. Total RNA was extracted from gonads, intestines, and coelomocytes of juvenile urchins, and samples were sequenced using MiSeq Illumina technology. A total of 91,119,300 paired-end reads were *de novo* assembled, 185,239 transcripts produced, and a reference transcriptome created with 38.8% GC content and an N50 of 1769 bp. Gene ontology analysis revealed notable differences in the expression profiles between gonads, intestines, and coelomocytes, allowing the detection of transcripts associated with specific biological processes and KEGG pathways. These data were validated using 12 candidate transcripts by real-time qPCR. This dataset will provide a valuable molecular resource for *L. albus* and other species of sea urchins.

## 1. Introduction

The *Loxechinus albus* (Molina, 1782), or edible red sea urchin, is an echinoderm species of the Chilean and Peruvian coasts, distributed along ca. Cape Horn, Chile (56°70′ S) to the Isla Lobos de Afuera, Peru (6°53′ S) [1]. The worldwide demand for high-quality gonads of this sea urchin has addressed a vast overexploitation of its natural populations [2]. Harvesting of *L. albus* represents the major sea urchin fishery among world urchin fisheries [3]. The aquaculture of this species, involving the rearing tank production of larvae, juvenile, and later fattening in natural environments, are important approaches to aquaculture diversification in Chile and to restore the overexploited coastal areas [4].

One of the main difficulties in the study of biological and molecular mechanisms associated with the farming of this species is the limited genomic information available [5,6]. In this context, transcriptome sequencing is useful to identify genes participating certain biological processes when genomic data are not available [7]. This analysis allows a broad comprehension of molecular mechanisms involved in biological processes from data on predicted function of genes [8]. Progress in the characterization of the transcriptome in commercial sea urchins is achievable due to advances in next-generation sequencing (NGS) technologies. NGS has allowed the research of sea urchin transcriptomes and other non-model species in brief periods of time at a low cost [9,10,11]. The molecular information achieved has provided significant value regarding the physiological responses to adaptation in a variety of commercial sea urchins under fluctuating environmental conditions [12,13].

At this time, the existing information on *L. albus* biology is limited and is related to with oxidative metabolism [14], growth patterns [15], the performance of early juveniles under food type and feeding frequency [16], and cryopreservation of embryos and larvae [17]. However, biological studies with molecular bases carried out in this species are scarce, mainly due to the low amount of genomic information available [11,18]. Although some advances have been made in the transcriptome characterization and mitogenome of this species in recent years, the low coverage of the technology used, as well as the use of gonads as the only target tissue, has limited the obtainment of a high-quality reference transcriptome [5,6,9,19]. Therefore, we present here the first annotated transcriptome of juvenile edible red sea urchin using NGS technologies based on three critical tissues for physiological homeostasis of echinoderms and the expression analysis of the transcripts present in each tissue: (i) gonads, involved in reproduction and exportation product for aquaculture, (ii) intestine, involved in food digestion and nutrient uptake, and (iii) coelomocytes, involved mainly in immune surveillance and inflammatory process. The transcriptome data obtained here will provide a reference for molecular studies in the farming of *L. albus* and other sea urchin species.

## 2. Materials and Methods

### 2.1. Experimental Design and Sampling

*Loxechinus albus* specimens were obtained from the Centro de Investigación Marina de Quintay (CIMARQ; 33°13′ S, 71°38′ O, Valparaiso, Chile). Briefly, fertilization was performed using a pool of gametes from four females and four males stimulated to spawn by injection of 3 mL of 0.5 M KCl. The embryos generated were cultured in 200 L larval rearing containers and larvae developed were fed with *Chaetoceros gracilis* microalgae. The larvae were grown in 50 L tanks in filtrated and aerated seawater and then preconditioned to settle in post-larval condition. Juvenile sexually immature sea urchins were maintained under natural conditions (13 ± 1 °C) in the spring season. The sea urchins were three years old and weighed 30 ± 5 g. The animals were fed macroalgae ad libitum (*Lessonia sp., Macrocystis sp., Durvillea sp.*). A total of 10 sea urchins were selected, dissected, and three different tissues were collected: intestines, gonads, and coelomocytes. Intestines were cleaned with phosphate buffer solution (PBS 1×) before storage. In immature gonads, germ cells were undifferentiated, revealing no sex differentiation. The coelomic fluid was collected by cutting the peristomal membrane, mixed with anticoagulant (20 mM Tris–HCl, 0.5 M NaCl, and 30 mM EDTA; pH 7.4), centrifuged for 5 min at 5000× *g*, and then coelomocyte pellet was collected. Samples were rapidly frozen in liquid nitrogen and deposited at −80 °C until use.

### 2.2. Isolation of RNA and Sequencing

Total RNA was obtained using columns of the RNeasy Mini Kit (Qiagen, Austin, TX, USA). The genomic DNA from RNA samples with removed by DNase I treatment. RNA was quantified by fluorometry using a Qubit 2.0 Fluorometer (Life Technology, Carlsbad, CA, USA), and the integrity of RNA was measured using the Fragment Analyzer (Analytical Advanced Technologies, Ames, IA, USA). Total RNA from five sea urchins were pooled in equal quantities by tissue, in duplicate, and then used to mRNA libraries construction. These libraries were generated by the TruSeq RNA Sample Preparation Kit v2 (Illumina, San Diego, CA, USA). Finally, libraries were sequenced (2 × 250 bp) utilizing the MiSeq technology (Illumina) at the Center for Plant Biotechnology (Universidad Andrés Bello, Santiago, Chile). The raw reads of the present study were uploaded to the NCBI SRA database under BioProject PRJNA475570, with accession number SRP150640.

### 2.3. Processing of Raw Data, De novo Assembly, and Validation of Assembly

First, the raw sequence reads were quality checked using FASTQC software. Adapters were removed, and raw data were trimmed using FlexBar [20] with Phred scores below 38 and 250 bp reads. The *de novo* transcriptome was assembled using all libraries (two libraries per tissue) with the Trinity program using default parameters [21]. Transcripts were filtered based on the minimal number of mapped reads with the Corset program using default parameters [22]. To evaluate *de novo* assembly integrity, the assembled transcriptome by Benchmarking Universal Single-Copy Orthologs (BUSCO) was compared against the OrthoDBv9 database (Vertebrata and Eukaryota) to identify orthologous genes that were highly conserved [23].

### 2.4. Functional Annotation and Analysis of Differentially Expressed Transcripts

For transcriptome annotation, a search in BlastX against the UniProt (https://www.uniprot.org/blast/; accessed 18 March 2021), Nonredundant (NR; https://blast.ncbi.nlm.nih.gov/Blast.cgi; accessed 18 March 2021), and Clusters of orthologous groups for eukaryotic complete genomes (COG; https://www.ncbi.nlm.nih.gov/research/cog; accessed 18 March 2021) databases was performed. The Blast2GO program was employed to acquire gene ontology (GO) annotation [24], and the WEGO application [25] was used to carry out GO functional classification for all transcripts. Recognition of differentially expressed transcripts (DETs) from the gonads, intestines, and coelomocytes were realized using Bowtie by mapping against the assembled *L. albus* transcriptome [26]. The RSEM software was used to assess expression values of fragments per kilobase million (FPKM) [27]. EdgeR was employed to determine differential expression between intestine vs. gonad, coelomocytes vs. intestine, and coelomocytes vs. gonads [28]. Transcripts detected with false discovery rate (FDR)-corrected *p* values < 0.001 and absolute values of fold-change > 4.0 were incorporated in the GO and Kyoto encyclopedia of genes and genomes (KEGG) enrichment analyses. 

### 2.5. Gene Ontology and KEGG Enrichment Analysis

The DETs were examined against the DAVID resource [29] and then categorized based on GO terms for molecular functions, biological processes, cellular components, and KEGG pathways. To determine a relationship between the DAVID background and *L. albus* DETs, a search in BLASTx was performed against *Strongylocentrotus purpuratus* Ensembl proteins for major matches with the *L. albus* transcriptome. Ensembl Gene IDs of *S. purpuratus* were acquired from the resultant Ensembl protein entries. Custom IDs set were selected for DAVID analysis as the “Background” Standard settings for ease (0.1) and gene count (2). The cut off *p* value used for molecular functions and cellular components was 1 × 10^−3^, and for biological processes was 1 × 10^−6^.

### 2.6. Validation of RNA-Seq by Real-Time qPCR

All quantitative real-time polymerase chain reaction (qPCR) assays were performed according to MIQE recommendations [30]. Total RNA isolation from gonads, intestines, and coelomocytes was realized using columns of RNeasy Mini Kit (Qiagen). RNA quantification was measured by NanoDrop technology with an Epoch Multivolume Spectrophotometer System (BioTek, Winooski, VT, USA). For complementary DNA (cDNA) synthesis, only RNA with an A260/280 ratio between 1.9 and 2.1 was selected. This procedure was performed using 1 μg of RNA by QuantiTect Reverse Transcription Kit (Qiagen), eliminating first genomic DNA with the wipeout buffer included and then reverse transcribed into cDNA at 42 °C for 30 min. qPCR reaction was made using Brilliant II SYBR Green QPCR Master Mix (Agilent Technologies, Santa Clara, CA, USA), a 1/dilution of cDNA, and 5 µM primers (Supplementary Table S1). Real-time PCRs were run on an Mx3000P qPCR System (Agilent Technologies) in triplicate. The PCR amplification program included 95 °C for 10 min; 40 cycles of 95 °C for 30 s, Tm for 30 s, 95 °C for 32 s, and 72 °C for 30 s. The QGene application was utilized to analyze gene expression [31] and data were normalized with 18S ribosomal subunit RNA as housekeeping gene.

### 2.7. Statistical Analysis

All statistical analyses were performed using GraphPad Prism v8.0 (GraphPad Software, La Jolla, CA, USA). The generated data were presented as the mean ± standard error of the mean (SEM). A one-way analysis of variance (ANOVA) followed by the Bonferroni post hoc test was used to differentiate the means between groups. A value of *p* < 0.001 was accepted as significative data. Finally, correlation analysis between real-time qPCR and RNA-seq were evaluated by multiple linear regression through *p* value and coefficients of determination (R^2^). 

## 3. Results

### 3.1. Raw Data Sequencing, De novo Assembly Transcriptome and Functional Annotation

**Figure 1:**
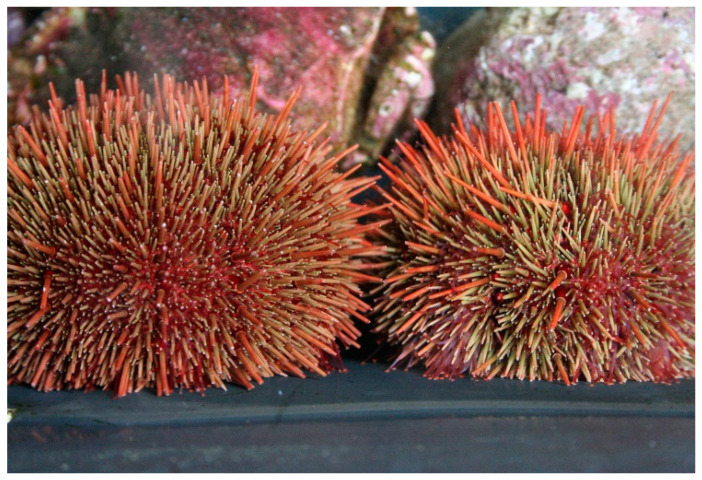
Juvenile edible red sea urchins (*Loxechinus albus*).

The cDNA libraries were generated from intestines, gonads, and coelomocytes of pooled edible red sea urchins (Figure 1).

**Figure 2:**
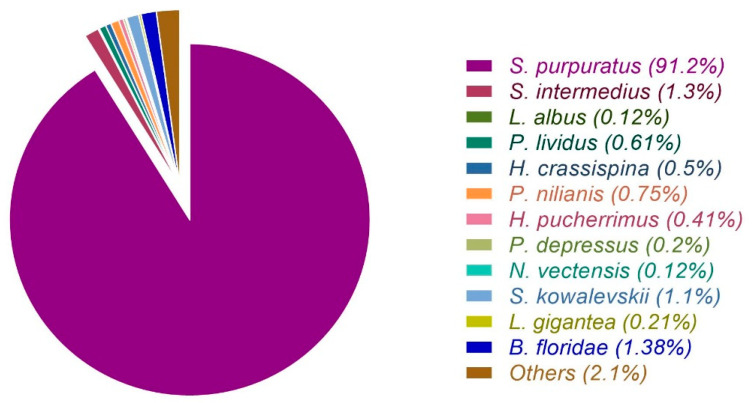
A species-based BlastX comparative analysis revealed the major match with *Strongylocentrotus purpuratus*.

The Illumina MiSeq sequencing produced 95,745,640 paired end reads of cDNA library replicates for each tissue (Table 1). The obtained raw data were trimmed by eliminating adapters, contaminant sequences and filtering base pairs with low-quality, the high-quality reads were reduced to 91,119,300 base pairs (Table 1). The Trinity software was used to *de novo* assembly using all libraries, resulting in 278,803 transcripts. The high-quality reads were mapped against the transcriptome generated and reduced by Corset software. The newly reduced transcriptome had 185,239 transcripts, with N50 = 1769 bp and GC% = 38.81 (Table 1). The length distribution of the obtained transcripts was detailed in Supplementary Figure S1. The BUSCO database was selected to compare the assembled transcriptome, which includes information of orthologous genes highly conserved. The assembly found 248 BUSCO genes of the eukaryotic core gene, 222 complete (89.5%), 8 fragmented (3.2%), and 18 missing genes (7.3%). The annotation of *L. albus* transcripts was carried out by BlastX searches against the non-redundant (NR), UniProt, and cluster of orthologous groups (COG) databases. The statistics of similarity and target species of all transcripts are shown in Figure 2, revealing that 91.2% of sequences matched *S. purpuratus*.

**Figure 3:**
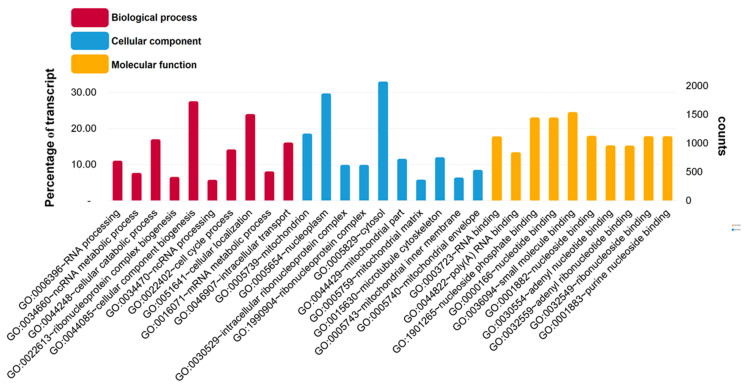
GO functional classification assigned the most percentage of the annotated transcripts to cellular component biogenesis term for biological process; cytosol term for cellular component; and small molecule binding term for molecular function, respectively. Analysis was carried out with the WEGO program for the edible sea urchin (*L. albus*) reference transcriptome.

The functional annotation of transcripts was realized using the Blast2GO platform, applying a GO term search through transcripts with BLAST hits matched against NR database. We detected significant similarity with a total of 57,106 (31%) transcripts. The GO analysis revealed 38,265 GO outcomes for biological processes (20.2%), 36,046 for molecular functions (19.1%) and 35,909 GO for cellular components (19.0%). A significant proportion of the annotated transcripts in biological process were assigned to cellular component biogenesis (GO:0044085) and cellular localization (GO:0051641) (Figure 3). 

**Figure 4:**
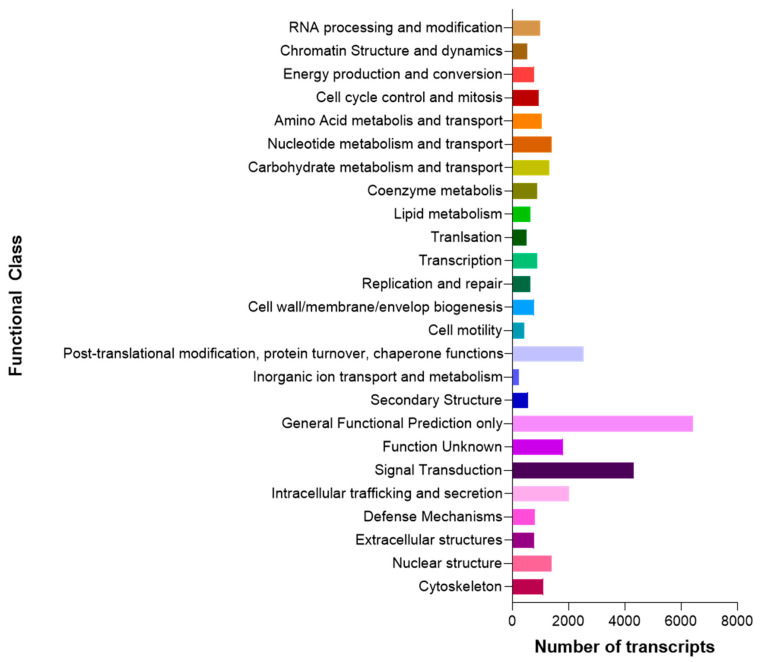
The General Functional Prediction only was the most represented category for the 25 cluster of orthologous groups (COG) functional classification in the *L. albus* reference transcriptome.

For cellular components and molecular functions, several annotated transcripts were allocated to cytosol (GO:0005829) and nucleoplasm (GO:0005654); and nucleotide binding (GO:0000166), nucleoside phosphate binding (GO:1901265), and small molecule binding (GO:0036094) terms, respectively (Figure 3). Based on sequence homology, 32,231 sequences were classified into 25 functional categories (Figure 4). The most represented categories were General Functional Prediction only, followed by Signal Transduction. These results indicate that we generated a reference transcriptome for the edible sea urchin based on 91,119,300 high-quality reads that assembled *de novo* into 185,239 transcripts with an N50 of 1769 bp and 38.8% GC content.

### 3.2. Assessment of Differentially Expressed Transcripts

**Figure 5:**
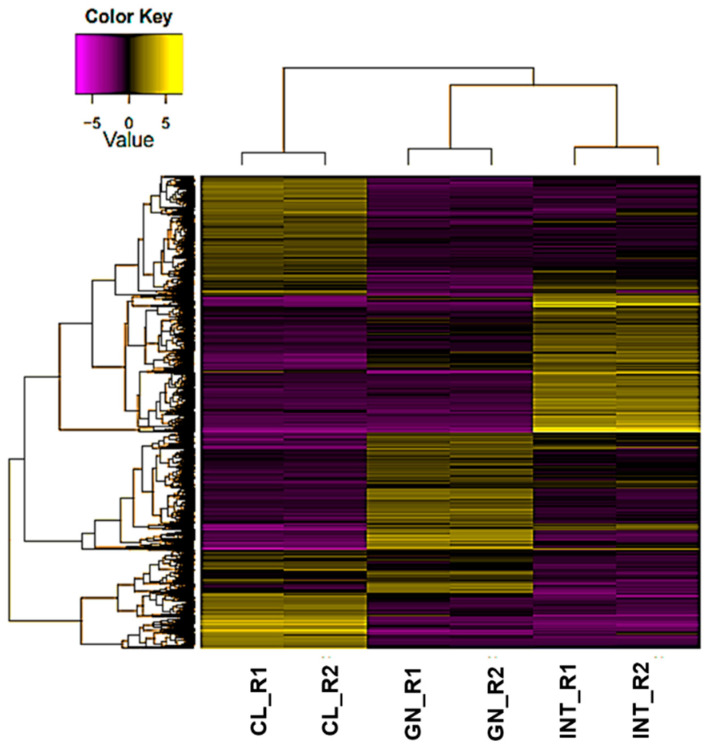
The heat map showed differentiated clustering of expressed transcripts according to respective expression values across tissues. Parameters: fold change (absolute values > 4.0) and FDR corrected *p* value (*p* < 0.001). Abbreviations: CL: coelomocyte, INT: intestine, GN: gonad. R1: Replicate 1, R2: Replicate 2.

About ~95.0% of the reads were mapped to the assembled transcriptome by Bowtie, and then RSEM and EdgeR were used to estimate the expression values and to calculate differential expression transcripts (DETs) between tissues, respectively. Transcripts with false discovery rate (FDR)-corrected *p* values < 0.001 and absolute fold-change values > 4.0 were defined as DETs. The mapping of *L. albus* transcriptome indicated that 26,864 transcripts were differentially expressed among coelomocytes, intestine, and gonad. These DETs were clustered using hierarchy by comparisons between patterns of gene expression (Figure 5). 

**Figure 6:**
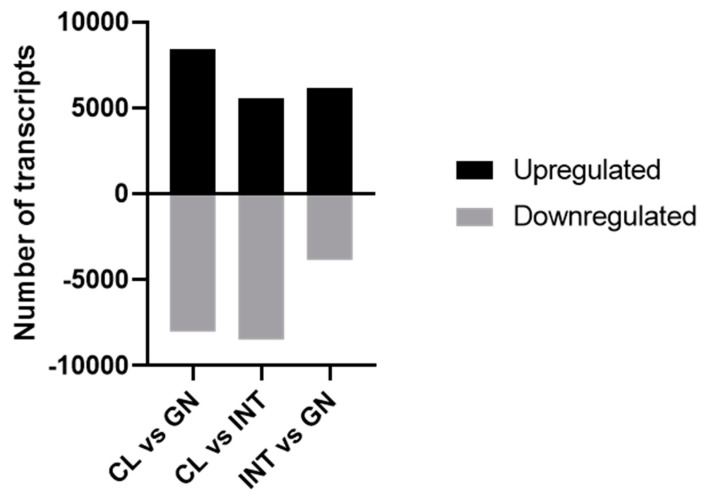
Paired comparisons of differentially expressed transcripts between tissues of *L. albus*. Upregulated and downregulated transcripts measured are indicated by black and gray columns, respectively. Parameters: fold change (absolute values > 4.0) and FDR corrected *p* value (*p* < 0.001). Abbreviations: CL: coelomocyte, INT: intestine, GN: gonad.

The correlation analysis of heat map clustered samples together in the same group, representing a high reproducibility of RNA-seq data among replicates (Supplementary Figure S2). To identified DETs, tissues were exposed to a succession of paired comparations (Figure 6): In coelomocytes vs. gonad, 16,406 DETs were identified (8394 upregulated and 8012 downregulated); in coelomocytes vs. intestine, 14,025 DETs were identified (5558 upregulated and 8467 downregulated); intestine vs. gonad, 10,015 DETs were identified (6179 upregulated and 3836 downregulated). 

**Figure 7:**
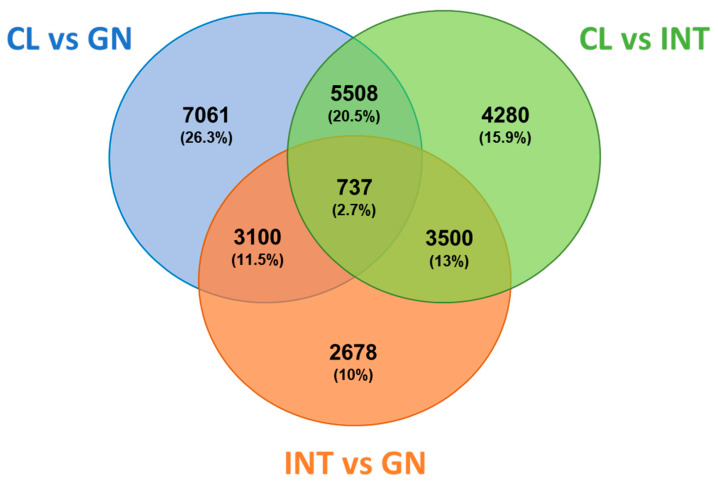
Differentially expressed transcripts between gonads, intestines, and coelomocytes of the edible sea urchin. Venn diagram of the total differentially expressed transcripts in *L. albus*. Each color indicates the comparison between tissue and the numbers of genes that were differentially expressed. Parameters: fold change (absolute values > 4.0) and FDR corrected *p* value (*p* < 0.001). Abbreviations: CL: coelomocyte, INT: intestine, GN: gonad.

**Figure 8:**
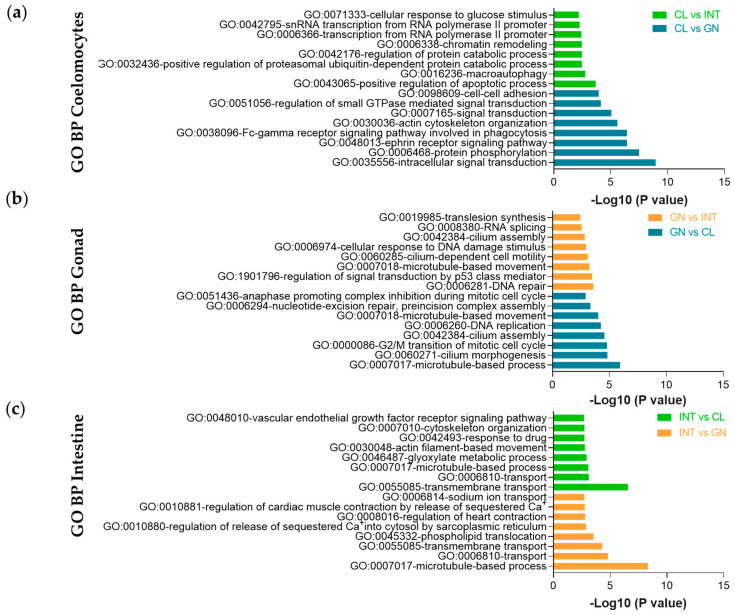
The Top-16 Gene Ontology biological process (BP) enrichment of up-regulated transcripts from *L. albus* tissues. (**a**) In coelomocytes compared to intestine (CL vs. INT), the most enriched term was positive regulation of apoptotic process, and compared to the gonad (CL vs. GN) was intracellular signal transduction; (**b**) in the gonad compared to intestine (GN vs. INT), the most enriched term was DNA repair, and compared to coelomocytes (GN vs. CL) was microtubule-based process; (**c**) in intestine compared to coelomocytes (INT vs. CL) the most enriched term was transmembrane transport, and compared to the gonad (INT vs. GN) was microtubule-based process.

The analysis of Venn diagram revealed that 737 DETs (2.7%) were commonly expressed among the three tissues (Figure 7). Also, 7061 DETs (26.3%) were specifically expressed between coelomocytes and gonad, specific 4280 DETs (15.9%) among coelomocytes and intestine, and 2678 DETs (10%) between intestine and gonad. A complete list of the DETs for each tissue was included in Supplementary Table S2. The tissue-specific DETs were examined in DAVID resource and categorized as biological processes, molecular functions, and cellular components. The coelomocyte DETs were considerably enriched in positive regulation of apoptotic process (GO:0043065) and intracellular signal transduction (GO:0035556) for biological processes (Figure 8a). The most enriched GO terms for molecular functions and cellular components were assigned to poly(A) RNA binding (GO:0044822) and ATP binding (GO:0005524), and cytosol (GO:0005829), respectively (Supplementary Figure S3). In contrast, DNA repair (GO:0006281) and microtubule-based process (GO:0007017) were the major enriched biological processes in gonads (Figure 8b). The main transcripts assigned to molecular functions and cellular components were microtubule motor activity (GO:0003777) and ATP binding (GO:0005524) GO terms, and dynein complex and cilium (GO:0005929) GO terms, respectively (Supplementary Figure S4). For biological processes, transmembrane transport (GO:0055085) and microtubule-based process (GO:0007017) were the most enriched in intestinal DETs (Figure 8c). The majority assigned transcripts for molecular functions and cellular components were GTP binding (GO:0005525) and motor activity (GO:0003774), and extracellular exosome (GO:0070062) and membrane (GO:0016020), respectively (Supplementary Figure S5).

The analysis of KEGG pathway indicated that various transcripts related to cAMP signaling pathway, Platelet activation, and Neurotrophin signaling pathway were overrepresented in coelomocytes vs. intestine analysis (Table 2). Other relevant pathways represented in coelomocytes vs. gonads correspond to Fc gamma R-mediated phagocytosis, Platelet activation, and Pathogenic Escherichia coli infection (Table 2). In gonad vs. intestine, the Purine metabolism was the main pathway overrepresented, followed by the Spliceosome and Huntington’s disease (Table 3). We also found that Biosynthesis of antibiotics, Gap junction, and Fatty acid degradation were highly represented in gonads vs. coelomocytes (Table 3). Regarding the main signaling pathways overrepresented in intestine (intestine vs. coelomocytes), we highlight adherens junction, Pathogenic Escherichia coli infection, and ABC transporters (Table 4). Finally, the comparison of intestine vs. gonad confirmed that pathways such ABC transporters and Pathogenic Escherichia coli infection were relevant overrepresented pathways (Table 4). In summary, we detected 26,864 transcripts were differentially expressed among the three tissues, which GO analysis revealed several processes and pathways that were expressed in common and, most importantly, tissue specific.

### 3.3. Transcriptomic Data Validation

**Figure 9:**
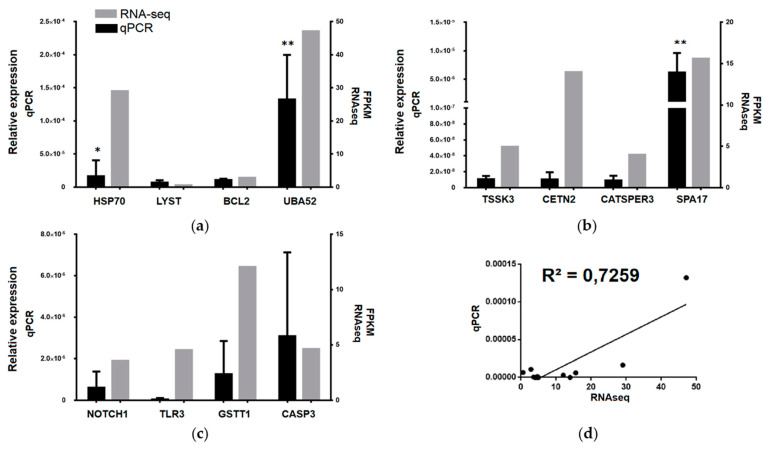
The quantitative real-time PCR validation of differentially expressed transcripts was highly correlated to RNA-seq. (**a**) coelomocyte; (**b**) gonad; (**c**) intestine; and (**d**) statistical correlation analysis. Expression fold changes measured by RT-qPCR and RNA-seq are indicated by black and gray columns, respectively. Abbreviations: heat shock protein 70 kDa 1 A (HSP70), lysosomal trafficking regulator (LYST), B-cell lymphoma 2 (BCL2), ubiquitin A-52 residue ribosomal protein fusion product 1 (UBA52), testis-specific serine/threonine-protein kinase 3 (TSSK3), centrin 2 (CETN2), cation channel sperm associated 3 (CATSPER3), sperm surface protein 17 (SPA17), notch homolog 1 (NOTCH1), toll-like receptor 3 (TLR3), glutathione s-transferase theta 1 (GSTT1), and caspase 3 (CASP3). * *p* < 0.05, ** *p* < 0.01.

We used real-time qPCR to validate RNA-seq and assay upregulated transcripts in each tissue compared to the others. According to *p* values and fold enrichments indicated in GO analysis, we selected four transcripts from coelomocytes: heat shock protein 70 kDa 1 A (HSP70), lysosomal trafficking regulator (LYST), B-cell lymphoma 2 (BCL2), and ubiquitin A-52 residue ribosomal protein fusion product 1 (UBA52) (Figure 9a). Four transcripts from gonad were identified: testis-specific serine/threonine-protein kinase 3 (TSSK3), centrin 2 (CETN2), cation channel sperm associated 3 (CATSPER3), and sperm surface protein 17 (SPA17) (Figure 9b). Four transcripts from intestine were identified: notch homolog 1 (NOTCH1), toll-like receptor 3 (TLR3), glutathione s-transferase theta 1 (GSTT1), and caspase 3 (CASP3) (Figure 9c). The transcript expression revealed a high statistical correlation between RNA-seq and qPCR measuring (R^2^ = 0.7259, *p* < 0.001) (Figure 9d). The data indicate that high correlation between RNA-seq and qPCR was obtained, validating the selected transcripts.

## 4. Discussion

In this study, the transcriptome of the edible red sea urchin (*L. albus*) was sequenced and annotated by using NGS technology. This is the first report on the RNA sequencing, transcriptome assembly, and functional annotation from juvenile *L. albus* based on three different tissues: intestines, gonads, and coelomocytes. This transcriptome contains 185,239 transcripts with similar features to existing transcriptomes of other sea urchins. For instance, the GC content of *L. albus* (38.8%) has a similar value in relation to the sea urchin transcriptomes of *Evechinus chloroticus* (39.0%) [32], *Sterechinus neumayeri* (38.6%) [33], *Strongylocentrotus intermedius* (39.6%) [34], and *Mesocentrotus nudus* (39.9%) [35]. However, our results exhibit a GC content slightly lower than values reported for the testis transcriptome of *L. albus* (40.4%) [6]. This small difference may be attributed to the tissue used in the study (mature gonad), as well as the sequencing technology (Roche 454 GS-FLX Titanium). This group obtained 1062,716 raw reads with a mean length of 309.8 bp, generating a reference transcriptome of 42,530 transcripts with an N50 of 645 bp [6], in contrast to our study, which presents 91,119,300 paired end reads with a mean length of 250 bp, generating a reference transcriptome of 185,239 transcripts with an N50 of 1769 bp.

A detailed analysis of tissue expression reveal that 26,864 transcripts are differentially expressed among the three tissues. Coelomocytes are the cells responsible for immunity in sea urchins, of which 50% to 70% are motile cells with a high energy requirement and are considered equivalent to human macrophages, which are cellular immune system components with high catabolic activity and are part of the innate immune response involved in pathogen digestion and autophagy [36,37]. Besides coelomocytes are predominantly in the coelomic fluid, they also function as wandering cells and infiltrate all tissues [36]. Consequently, the differentially expressed transcripts in the coelomocytes are mainly associated with biological processes, such as positive regulation of apoptotic process and intracellular signal transduction, and KEGG pathways associated with Platelet activation, Fc gamma R-mediated phagocytosis, and Pathogenic *Escherichia coli* infection. Studies in sea urchin coelomocytes transcriptomes describe similar observations. In *S. intermedius*, the expression of 546 unique transcripts in coelomocytes is associated with lysozyme, lectin, pattern recognition receptors (PRRs), and the complement system [38]. In a related study carried out in coelomocytes of *Arbacia lixula*, the expression of transcripts is associated to lipid metabolism and the immune response [39]. In addition, an RNA-seq analysis in coelomocytes reveal key functions of NOD-like receptor pathway and phagosomes in spotting diseased *S. intermedius* [40]. Recently, the immune response of *L. albus* coelomocytes by poly I:C, bacterial lipopolysaccharides (LPS), and temperature reveal a dynamic expression of TLR genes (*tlr3* including), as well as *strongylocin-1* and *strongylocin-2* [18].

Among the transcripts identified with a high expression in coelomocytes and validated by RT-qPCR stand out the heat shock protein 70 kDa 1 A (HSP70), the lysosomal trafficking regulator (LYST), the B-cell lymphoma 2 (BCL2) and the ubiquitin A-52 residue ribosomal protein fusion product 1 (UBA52). HSP70 is a chaperone protein responsible for protein folding to protect cells against stressors or presenting antigens for immune response [41]. Interestingly, a recent study has shown that LPS can induce a stress response by increasing the protein levels of HSP70 in *Paracentrotus lividus* coelomocytes, suggesting a relevant role in the sea urchin immune response [42]. LYST plays a role in the transport of materials into structures called lysosomes, acting as recycling centers within cells [43]. Although there are no reports of the importance of this gene in sea urchin coelomocytes, in mammalian macrophages has been linked as a key regulator of membrane trafficking to inflammatory responses mediated by TLRs [44]. BCL2 is a member of protein regulators for cell death, through inhibition of apoptosis [45]. The participation of BCL2 as an important mediator of the immune response in marine organisms has recently been described in *Apostichopus japonicus* challenged with *Vibrio splendidus* [46]. The UBA52 gene encodes to 60S ribosomal protein L40 (RPL40) and, together with ubiquitin, has a main function of targeting proteins for degradation by the 26S proteosome. Additionally, UBA52 can regulate gene expression, chromatin structure, and the stress response [47]. Although there are no reports of the relevance of UBA52 in the immune response of sea urchin coelomocytes, its participation in the immune response of higher vertebrates has been described [48]. These observations suggest a permanent activity of protein catabolism in sea urchin coelomocytes, as sentinel organisms of the immune response.

In sea urchins, the gonads are considered a dual organ since they regulate reproduction and nutrient storage [49]. Both functions are carried out by two specific cell populations, somatic and germ cells, the latter also called nutritive phagocytes [50]. Several transcriptomic analyses have been described in gonads of edible sea urchin species, obtaining results in agreement with ours. Particularly, a *de novo* assembly of *M. nudus* gonad transcriptome, key genes associated with biological processes such lipid metabolism, and the biosynthesis of polyunsaturated fatty acids have been identified [35]. Furthermore, a comparative analysis between reproductive tissues of this specie, reveal upregulated GO categories related to energy generation in the testis and negative regulation of nucleotide metabolism in the ovary [39]. Additionally, a transcriptomic analysis indicates important differences in the expression of genes of *M. nudus* related to the biosynthesis of polyunsaturated fatty acids and metabolism in relation to high-quality gonads, similar to our observations [34,51]. In addition, a gonadal transcriptomic of *M. nudus* present candidate sex-related genes that could be involved in significant roles in spermatogenesis, oogenesis, and germ cell development [52]. More recently, integrated analyses of metabolomic and transcriptomic reveal key genes for metabolism and eicosapentaenoic acid biosynthesis in the sea urchin *S. intermedius*, identifying six accumulated metabolites and several differentially expressed genes associated with polyunsaturated fatty acids in the testis compared with the ovary [53].

The testis-specific serine/threonine-protein kinase 3 (TSSK3), centrin 2 (CETN2), cation channel sperm associated 3 (CATSPER3) and sperm surface protein 17 (SPA17) were upregulated specifically in gonad. TSSK3 is a protein involved in the development and maturation of male germ cell [54]. TSSK3 role as a key regulator in spermiogenesis of the Scallop *Argopecten irradians* has recently been described [55]. CETN2 is a member of calcium-binding proteins and is also a component of the centrosome [56]. However, there are no previous reports of its importance in the gonadal function of marine echinoderms. CATSPER3 is a sperm-specific ion channel that plays a central role for the successful fertilization, which involves sperm hyperactivation, acrosome reaction, and chemotaxis towards the egg [57]. In mammals, it has been determined that CATSPER3 is exclusively expressed in the testis, which should be validated in future analyzes in adult sea urchins [58]. SPA17 encodes a sperm surface zona pellucida binding protein. In mammals, SPA17 may be implicated in fertilization through zona pellucida attaching of the oocyte [59]. Members of this group of genes have shown an important participation in the maturation and fertilization of echinoderm eggs [60]. However, it is difficult to speculate if differentially expressed transcripts of gonads are relevant in the function of the testis or ovary, because the present study was performed with sexually immature juveniles *L. albus*.

Finally, the intestine is the organ responsible for the digestion of food [61]. In echinoderms, it also has a key role in the immune response, where it has been described that it presents a high number of sequences related to this process [62,63]. Consistently, we observe an overrepresentation of GO and KEGG categories similar to that observed in coelomocytes, showing high enrichment in the sequences related to SSCR and Cargo Receiver. This is consistent with the importance of the intestine in the immune response; therefore, it has been postulated that the control of normal flora or pathogen-associated gut response is responsible for the increase in the expression of the pattern recognition receptors NLR and TLR [64]. Similarly, we also detect an overrepresentation of lysosomal pathways, which was also has been found in the transcriptomic analysis in *S. intermedius* [38].

The notch homolog 1 (NOTCH1), toll-like receptor 3 (TLR3), glutathione s-transferase theta 1 (GSTT1) and caspase 3 (CASP3) were upregulated specifically in the intestine. NOTCH1 is a Type 1 transmembrane protein involves in numerous processes of development by regulating cellular fate. The Notch signaling pathway is highly conserved among species and regulates interactions between adjacent cells [65]. Interestingly, the importance of intestinal epithelial NOTCH1 as a protector for the development of colorectal adenocarcinoma in a murine model has been described [66]. TLR3 is a member of pattern recognition receptors that recognizes specific molecules of pathogens during innate immune response [67]. Recently our group described the upregulation of TLR3 expression in the coelomocyte response to bacterial LPS, poly I:C and temperature in *L. albus* [18]. GSTT1 is an enzyme that catalyzes the coupling of reduced glutathione to diverse hydrophobic and electrophilic compounds [68]. Remarkably, the glutathione S-transferase activity in the anterior portion of *P. lividus* intestine has been employed as biomarker of environmental contamination, revealing an important role in the homeostasis of this tissue [69]. Finally, CASP3 gene encodes for a cysteine-aspartic acid protease (caspase) that has a crucial role in the execution-phase of apoptosis [70]. This protein has been described as an essential element if the innate immunity of sea urchins [64]. These observations suggest the presence of coelomocytes in the sea urchin intestinal tract, as a fundamental part of the crosstalk between intestinal microbiota and the inflammatory response.

## 5. Conclusions

This is the first evidence on the RNA sequencing, *de novo* assembly, and functional annotation of the edible red sea urchin (*L. albus*) transcriptome, as well as the differential expression in the gonads, intestines, and coelomocytes. The *de novo* assembly produced 185,239 transcripts, creating a reference transcriptome with an N50 of 1769 bp and 38.8% GC content. Gene ontology analysis of transcripts revealed notable differences in the expression profiles between gonads, intestines, and coelomocytes, allowing the detection of transcripts associated with specific biological processes. In coelomocytes, DETs were mostly associated to positive regulation of apoptotic process and intracellular signal transduction. In the gonad, DETs were associated to DNA repair and microtubule-based process. In Intestine, DETs were associated to transmembrane transport and microtubule-based process. The dataset generated in this work contribute to enrich the molecular resources of *L. albus*, improvement futures biological studies of this species. The acquired information is also relevant to discovering novel candidate genes that could be employed to estimate the physiological condition of edible red sea urchins under aquaculture rearing.

## Figures and Tables

**Table 1:** Summary of transcriptome sequencing for the edible red sea urchin (*L. albus*) tissue and assembly statistics. bp: base pair.

Name	Number of Reads	Number of Reads after Trim
Coelomocyte	20,682,190	19,948,624
Coelomocyte (replicate)	16,865,448	15,699,186
Intestine	12,145,212	11,748,696
Intestine (replicate)	11,348,164	10,620,928
Gonad	18,495,858	17,901,954
Gonad (replicate)	16,208,768	15,199,912
Total	95,745,640	91,119,300
Transcriptome	*De novo* Assembly	After Filter
	(Trinity)	(Corset)
Total contigs	278,803	185,239
Average large contig (bp)	326	929
Coverage contig	708.32	-
%GC	38.2	38.81
N10 (bp)	5015	5328
N30 (bp)	2645	2945
N50 (bp)	1418	1769
Total bases	197,480,887	172,122,576

**Table 2:** The Top-16 KEGG pathways enrichment of up-regulated transcripts of coelomocytes in the edible red sea urchin (*L. albus*). In comparison to intestine, cAMP signaling pathway was the most overrepresented and Fc gamma R-mediated phagocytosis compared to gonad.

**Coelomocytes vs. Intestine**				
**KEGG Term (ID)**	**Number of Genes**	**Percentage of Genes**	**Fold Enrichment**	***p* Value**
cAMP signaling pathway (4024)	13	2.9%	2.4	8.1 × 10^−3^
Platelet activation (4611)	10	2.2%	2.8	9.3 × 10^−3^
Neurotrophin signaling pathway (4722)	9	2.0%	2.7	1.7 × 10^−2^
Regulation of lipolysis in adipocytes (4923)	6	1.4%	3.8	1.8 × 10^−2^
Proteoglycans in cancer (5205)	12	2.7%	2.2	2.1 × 10^−2^
Focal adhesion (4510)	12	2.7%	2.1	2.6 × 10^−2^
Insulin secretion (4911)	7	1.6%	3.0	2.9 × 10^−2^
cGMP-PKG signaling pathway (4022)	10	2.2%	2.3	3.0 × 10^−2^
**Coelomocytes vs. Gonad**				
**KEGG Term (ID)**	**Number of Genes**	**Percentage of Genes**	**Fold Enrichment**	***p* Value**
Fc gamma R-mediated phagocytosis (4666)	15	2.5%	4.9	1.7 × 10^−6^
Platelet activation (4611)	16	2.6%	3.6	7.0 × 10^−5^
Pathogenic *Escherichia coli* infection (5130)	10	1.6%	5.4	7.9 × 10^−5^
cGMP-PKG signaling pathway (4022)	17	2.8%	2.9	1.9 × 10^−4^
Gap junction (4540)	12	1.9%	3.7	3.3 × 10^−4^
Proteoglycans in cancer (5205)	19	2.0%	2.6	3.5 × 10^−4^
Focal adhesion (4510)	19	3.1%	2.5	4.9 × 10^−4^
Bacterial invasion of epithelial cells (5100)	11	1.8%	3.8	4.9 × 10^−4^

**Table 3:** The Top-16 KEGG pathways enrichment of up-regulated transcripts of gonad in the edible red sea urchin (*L. albus*). In comparison to intestine, purine metabolism was the most overrepresented and Biosynthesis of antibiotics compared to coelomocytes.

**Gonad vs. Intestine**				
**KEGG Term (ID)**	**Number of Genes**	**Percentage of Genes**	**Fold Enrichment**	***p* Value**
Purine metabolism (230)	10	2.9%	2.4	6.9 × 10^−3^
Spliceosome (3040)	8	2.3%	2.8	1.4 × 10^−2^
Huntington’s disease (5016)	9	2.6%	2.7	3.2 × 10^−2^
p53 signaling pathway (4115)	5	1.5%	3.8	4.0 × 10^−2^
Focal adhesion (4510)	9	2.6%	2.1	4.6 × 10^−2^
Metabolic pathways (1100)	32	9.2%	3.0	5.9 × 10^−2^
Pathogenic *Escherichia coli* infection (5130)	4	1.5%	2.3	7.5 × 10^−2^
Gap junction (4540)	5	1.7%	2.4	9.0 × 10^−2^
**Gonad vs. Coelomocytes**				
**KEGG Term (ID)**	**Number of Genes**	**Percentage of Genes**	**Fold Enrichment**	***p* Value**
Biosynthesis of antibiotics (1130)	20	4.5%	3.4	4.5 × 10^−6^
Gap junction (4540)	10	2.3%	4.1	6.2 × 10^−4^
Fatty acid degradation (71)	7	1.6%	6.1	8.9 × 10^−4^
Valine, leucine, and isoleucine degradation (280)	7	1.6%	5.4	1.6 × 10^−3^
Fatty acid metabolism (1212)	7	1.6%	5.3	1.8 × 10^−3^
Phagosome (4145)	12	2.7%	2.9	2.6 × 10^−3^
Metabolic pathways (1100)	47	10.6%	1.4	1.1 × 10^−2^
DNA replication (3030)	5	1.1%	5.1	1.6 × 10^−2^

**Table 4:** The Top-16 KEGG pathways enrichment of up-regulated transcripts of intestine in the edible red sea urchin (*L. albus*). In comparison to coelomocytes, adherens junction was the most overrepresented and ABC transporters compared to gonad.

**Intestine vs. Coelomocytes**				
**KEGG Term (ID)**	**Number of Genes**	**Percentage of Genes**	**Fold Enrichment**	***p* Value**
Adherens junction (4520)	13	2.1%	5.0	7.7 × 10^−6^
Pathogenic *Escherichia coli* infection (5130)	11	1.7%	5.9	1.2 × 10^−5^
ABC transporters (2010)	10	1.6%	6.2	2.2 × 10^−5^
Lysosome (4142)	16	2.5%	3.6	2.9 × 10^−5^
Metabolic pathways (1100)	68	10.7%	1.5	1.8 × 10^−4^
Gap junction (4540)	11	1.7%	3.4	1.2 × 10^−3^
Chemical carcinogenesis (5204)	9	1.4%	3.1	8.2 × 10^−3^
cAMP signaling pathway (4024)	15	2.3%	2.1	1.3 × 10^−2^
**Intestine vs. Gonad**				
**KEGG Term (ID)**	**Number of Genes**	**Percentage of Genes**	**Fold Enrichment**	***p* Value**
ABC transporters (2010)	11	1.9%	7.9	7.1 × 10^−7^
Chemical carcinogenesis (5204)	12	2.2%	4.8	3.4 × 10^−5^
Pathogenic *Escherichia coli* infection (5130)	9	1.6%	5.6	1.6 × 10^−4^
Lysosome (4142)	12	2.2%	3.2	1.3 × 10^−3^
Gap junction (4540)	10	1.8%	3.6	1.6 × 10^−3^
Galactose metabolism (52)	6	1.1%	6.4	2.1 × 10^−3^
Starch and sucrose metabolism (500)	6	1.1%	5.8	3.3 × 10^−3^
Amino sugar and nucleotide sugar metabolism (520)	7	1.3%	4.6	3.6 × 10^−3^

## Data Availability

The raw read sequences obtained from sequencing were deposited in the Sequence Read Archive (SRA) under BioProject PRJNA475570, with accession number SRP150640. The datasets generated and/or analyzed during the current study are not publicly available due to privacy or ethical restrictions but are available from the corresponding author upon reasonable request.

## Supplementary Materials

The following are available online at https://www.mdpi.com/article/10.3390/biology10100995/s1, Table S1: Primer sequences for qPCR assays, including amplicon size and PCR efficiencies for the assessed genes, Table S2: The complete list of differentially expressed transcripts in the edible sea urchin (*L. albus*), Figure S1: Length distribution of assembled transcripts, Figure S2: The heat map analysis of transcript expression across tissues in *L. albus* was highly correlated among replicates, Figure S3: The Top-16 Gene Ontology molecular function (MF) and cellular component (CC) enrichment of up-regulated transcripts from *L. albus* coelomocytes, Figure S4: The Top-16 Gene Ontology molecular function (MF) and cellular component (CC) enrichment of up-regulated transcripts from *L. albus* gonad, Figure S5: The Top-16 Gene Ontology molecular function (MF) and cellular component (CC) enrichment of up-regulated transcripts from *L. albus* intestine.
