# Supplementary material for: De novo Assembly and Analysis of Tissue-Specific Transcriptomes of the Edible Red Sea Urchin Loxechinus albus Using RNA-Seq
Source: Biology (Basel). 2021 Oct 2;10(10):995. doi: 10.3390/biology10100995 (PMC8533317; doi:10.3390/biology10100995)
Supplement: Supplementary file 1 [file biology-10-00995-s001.zip › suplementaryfigs1.pdf]

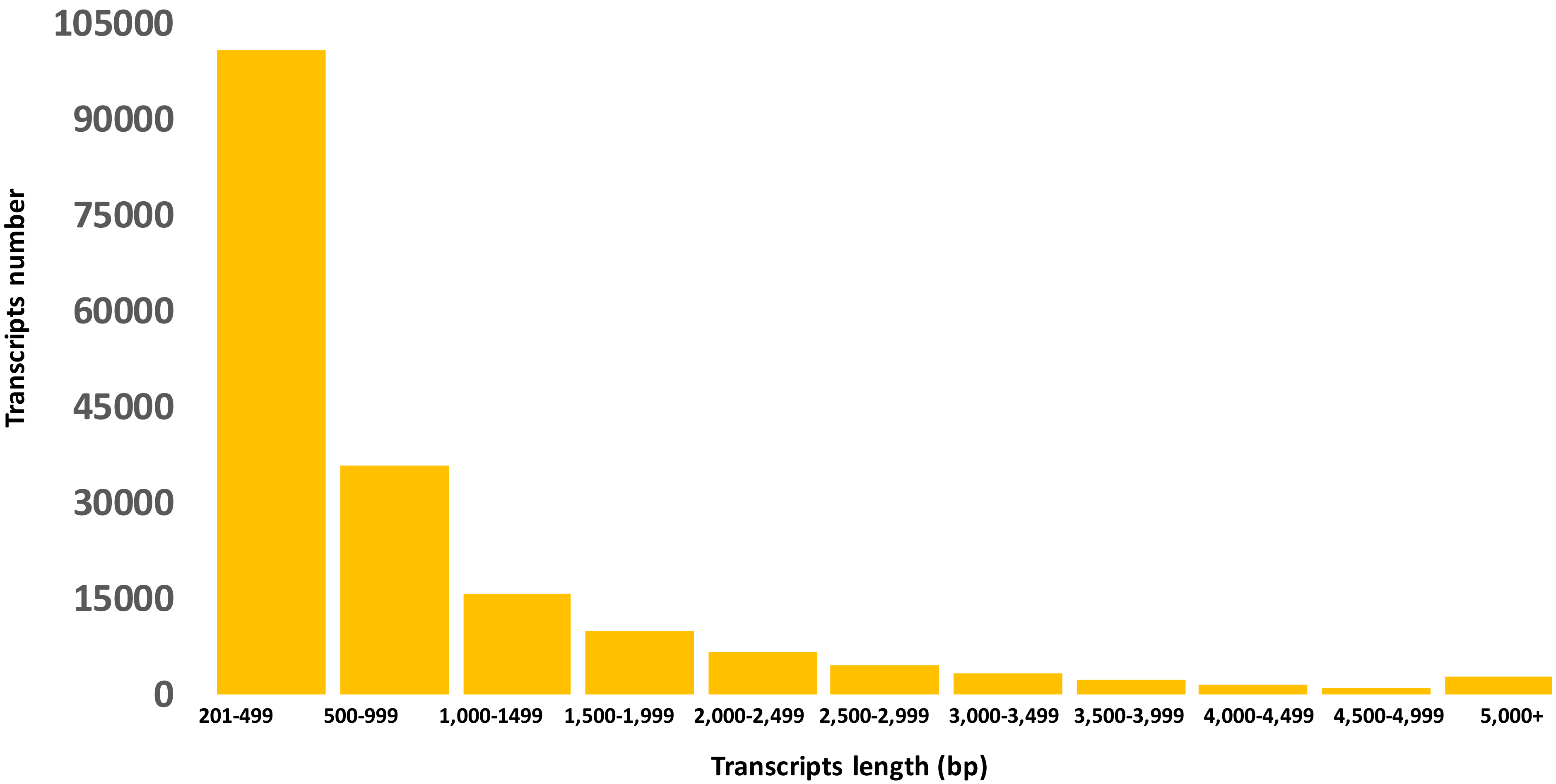

Figure S1: Length distribution of assembled transcripts. The sizes of the Trinity assembled transcripts were ranged from 200 to 5000 base pairs.
