# Supplementary material for: De novo Assembly and Analysis of Tissue-Specific Transcriptomes of the Edible Red Sea Urchin Loxechinus albus Using RNA-Seq
Source: Biology (Basel). 2021 Oct 2;10(10):995. doi: 10.3390/biology10100995 (PMC8533317; doi:10.3390/biology10100995)
Supplement: Supplementary file 1 [file biology-10-00995-s001.zip › supplementaryfigs2.pdf]

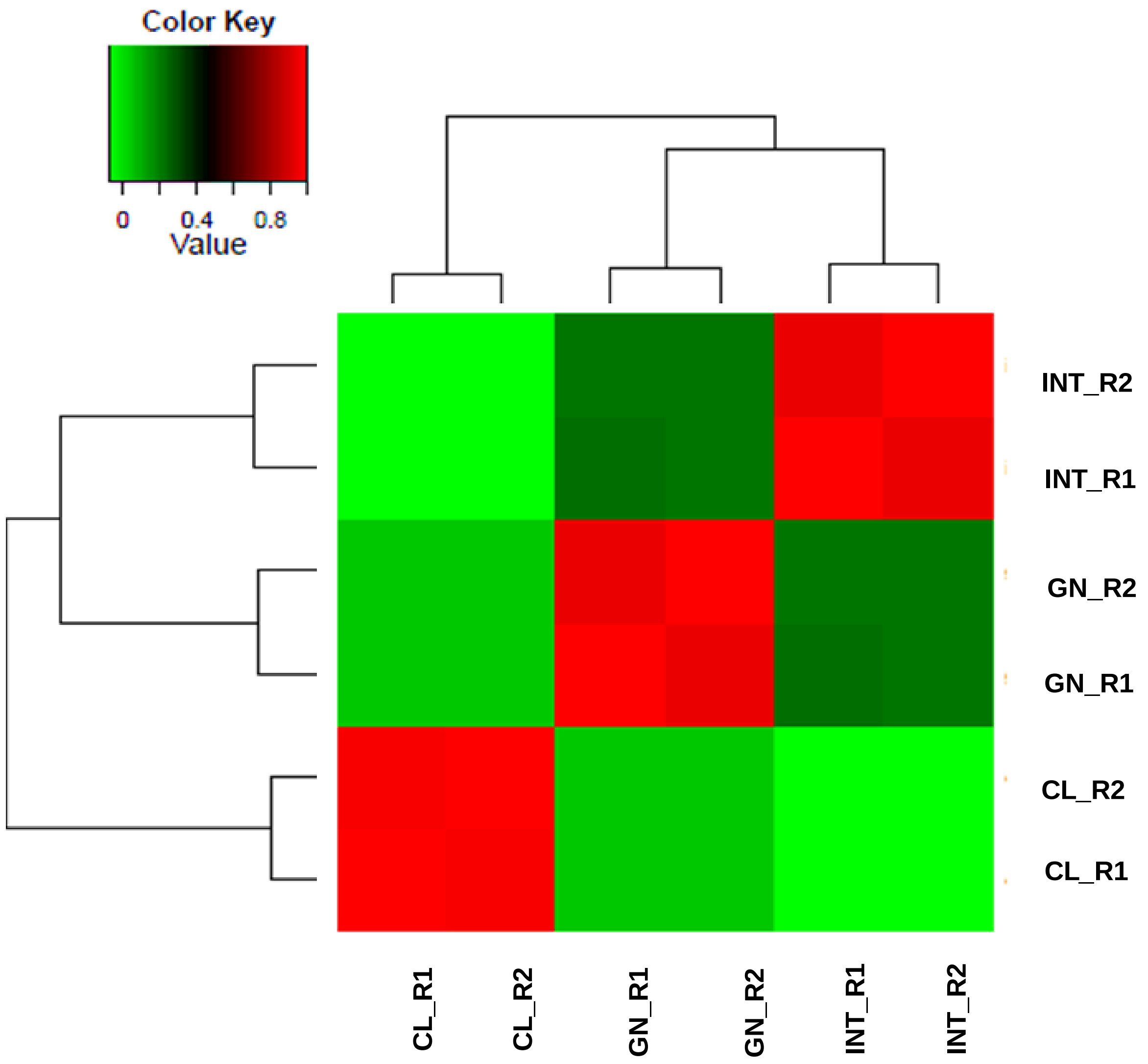

Figure S2: The heat map analysis of transcript expression across tissues in *L. albus* was highly correlated among replicates. INT_R1 and INT_R2 are replicates of intestines; CL_R1 and CL_R2 are replicates of coelomocytes; GN_R1 and GN_R2 are replicates of gonads.
