# Supplementary material for: De novo Assembly and Analysis of Tissue-Specific Transcriptomes of the Edible Red Sea Urchin Loxechinus albus Using RNA-Seq
Source: Biology (Basel). 2021 Oct 2;10(10):995. doi: 10.3390/biology10100995 (PMC8533317; doi:10.3390/biology10100995)
Supplement: Supplementary file 1 [file biology-10-00995-s001.zip › supplementaryfigs3.pdf]

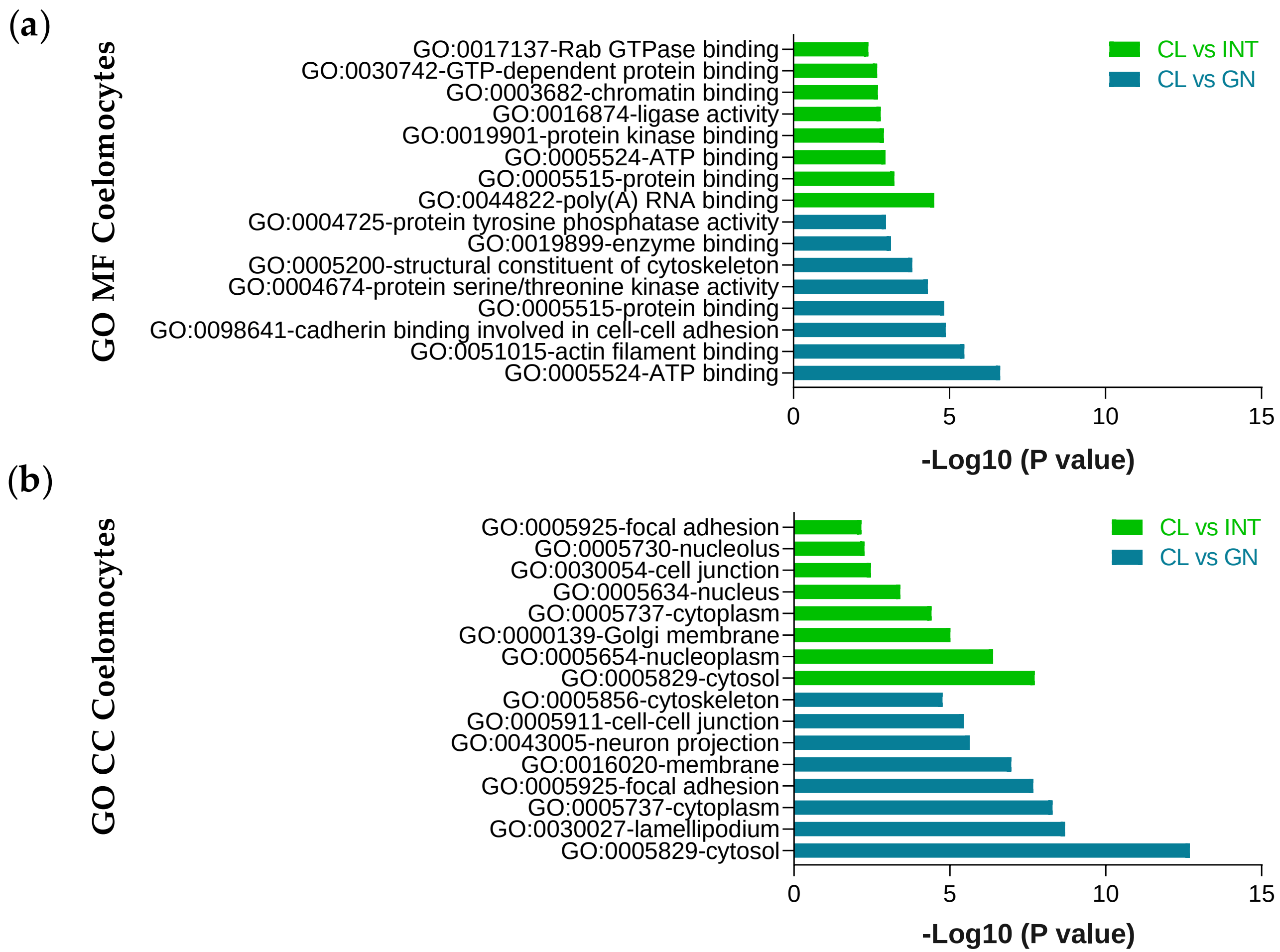

Figure S3: The Top-16 Gene Ontology molecular function (MF) and cellular component (CC) enrichment of up-regulated transcripts from *L. albus* coelomocytes. (**a**) In coelomocytes compared to intestine (CL vs INT), the most enriched term for MF was poly(A) RNA binding and compared to gonad (CL vs GN) was ATP binding; (**b)** Cytosol was the most represented term for CC.
